# The amino‐terminal domain of *Mycobacterium tuberculosis* ClpB protein plays a crucial role in its substrate disaggregation activity

**DOI:** 10.1002/2211-5463.12509

**Published:** 2018-09-15

**Authors:** Prajna Tripathi, Priyanka Parijat, Virendra Kumar Patel, Janendra K. Batra

**Affiliations:** ^1^ National Institute of Immunology New Delhi India; ^2^ Department of Biochemistry School of Chemical and Life Sciences Jamia Hamdard New Delhi India; ^3^ Present address: Randall Division of Cell and Molecular Biophysics King's College London UK

**Keywords:** Clp proteins, disaggregation, DnaK, heat shock proteins (HSP), *M. tuberculosis*, Rv0384c

## Abstract

*Mycobacterium tuberculosis* (*Mtb*) is known to persist in extremely hostile environments within host macrophages. The ability to withstand such proteotoxic stress comes from its highly conserved molecular chaperone machinery. ClpB, a unique member of the AAA+ family of chaperones, is responsible for resolving aggregates in *Mtb* and many other bacterial pathogens. *Mtb* produces two isoforms of ClpB, a full length and an N‐terminally truncated form (ClpB∆N), with the latter arising from an internal translation initiation site. It is not clear why this internal start site is conserved and what role the N‐terminal domain (NTD) of *Mtb* ClpB plays in its function. In the current study, we functionally characterized and compared the two isoforms of *Mtb* ClpB. We found the NTD to be dispensable for oligomerization, ATPase activity and prevention of aggregation activity of ClpB. Both ClpB and ClpB∆N were found to be capable of resolubilizing protein aggregates. However, the efficiency of ClpB∆N at resolubilizing higher order aggregates was significantly lower than that of ClpB. Further, ClpB∆N exhibited reduced affinity for substrates as compared to ClpB. We also demonstrated that the surface of the NTD of *Mtb* ClpB has a hydrophobic groove that contains four hydrophobic residues: L97, L101, F140 and V141. These residues act as initial contacts for the substrate and are crucial for stable interaction between ClpB and highly aggregated substrates.

**Abbreviations**
KJE: DnaK, DnaJ, GrpE
MDH: malate dehydrogenase
*Mtb*: *Mycobacterium tuberculosis*
NBD: nucleotide binding domain
NTD: N‐terminal domain
WT: wild‐type

To date tuberculosis (TB) remains the single largest infectious disease causing the highest mortality rate in humans worldwide. The ability of *Mycobacterium tuberculosis* (*Mtb*), the causative agent of TB, to survive under a plethora of stresses inside the host is an important aspect of its pathogenesis 1, 2. The airborne bacilli travel to the host alveoli where they rapidly become phagocytosed by alveolar macrophages and persist within the macrophages in the form of caseous granulomas. The environment encountered by persisters inside the granulomatous lesions is nutritionally deprived, inflamed and highly anoxic. Such stresses result in damage to the bacteria by causing their proteins to partially fold, misfold or even eventually aggregate 3. In response, the bacterial molecular chaperones become activated and make complexes with damaged proteins to revive them or to eliminate them by directing them to a peptidase partner. The network of molecular chaperones and proteases, which ensures cell survival under stress, is evolutionarily conserved in most bacteria and helps them achieve proteostasis by advancing either the reactivation or the degradation of unfolded, misfolded or aggregated proteins 3, 4. A special class of the chaperone network is the Clp (caseinolytic protease) proteins or Hsp100, which are a part of the AAA^+^ (ATPases associated with various cellular activities) superfamily. A distinct characteristic of this family of proteins is the presence of a nucleotide binding domain (NBD) (or AAA domain) which provides them with the ability to hydrolyze ATP and use its energy to generate the mechanical force required for restructuring the bound substrates 5. Clp proteins are further classified into two classes based on the number of nucleotide binding domains they possess: class I proteins, with two nucleotide binding domains, including ClpA, ClpB and ClpC, and class II proteins, containing only one NBD, including ClpX and ClpY. Some of these proteins, e.g. ClpA, ClpC and ClpX, each of which possesses unfolding activity, associate with a proteolytic partner, ClpP, to form proteasome‐like complexes that mediate the degradation of incorrectly folded proteins as well as the turnover of unwanted regulatory proteins and tagged proteins 6. However, extreme stress conditions can overload this otherwise robust degradation machinery, resulting in accumulation of protein aggregates. Under such harsh conditions, the bacterial cells enter into an energy saving mode that favors recycling of existing proteins over degradation and synthesis of new ones 4. The re‐utilization of aggregated proteins is facilitated by ClpB, which has a remarkable ability to pull out proteins from non‐native states by disaggregating them. ClpB is a unique member of the Clp family as instead of associating with any proteolytic subunit to exhibit degradation of substrates, it collaborates with another piece of chaperone machinery, the DnaKJE complex, and forms a network that specializes in resolubilization of proteins trapped in aggregates 7. The DnaK system functions upstream of ClpB in early steps of protein disaggregation and unfolding by helping in substrate recognition. With the help of its auxiliary chaperone, DnaJ, DnaK binds to the aggregates and subsequently recruits ClpB for interaction with substrate 8. Interaction of oligomeric barrel‐shaped ClpB with the exposed ends of substrate triggers ATP hydrolysis and promotes threading of the substrate through the central channel, eventually releasing the extended polypeptide from the exit pore whereafter it is free to refold 9, 10, 11. It is noteworthy that ClpB or DnaKJE individually exhibits only weak disaggregation activity 12.

ClpB manifests its optimal activity after attaining a hexameric barrel‐shaped ring conformation, which is the hallmark of the Clp family of proteins 13. A typical monomer of ClpB is about 95 kDa with multiple domains. In addition to the NBD common to Clp ATPases, ClpB has a unique middle domain inserted in the small domain of NBD1 (Fig. 1A). This domain is about 120 amino acids long and contains a bundle of four α‐helices that form a long coiled coil 14. The instability and flexibility of the M‐domain have been reported to be critical for ClpB disaggregation activity 15. Previous studies indicate that the N‐terminal region of ClpB forms a distinct domain that is loosely associated with the ClpB core and is not required for ClpB interactions with other co‐chaperones 16, 17. The N‐terminal domain (NTD) is a globular, helical domain that spans 1–148 amino acids and is linked with rest of the protein with a short unstructured linker. It has been shown that *Escherichia coli* ClpB has an alternate translation start site, coded by the GUG codon present at the 149th position of the protein sequence, which results in the production of two isoforms, a full length and an N‐terminally truncated ClpB proteins 18. Except for the lack of the NTD, the N‐terminally truncated isoform is structurally identical to the full length ClpB 19.

**Figure 1:**
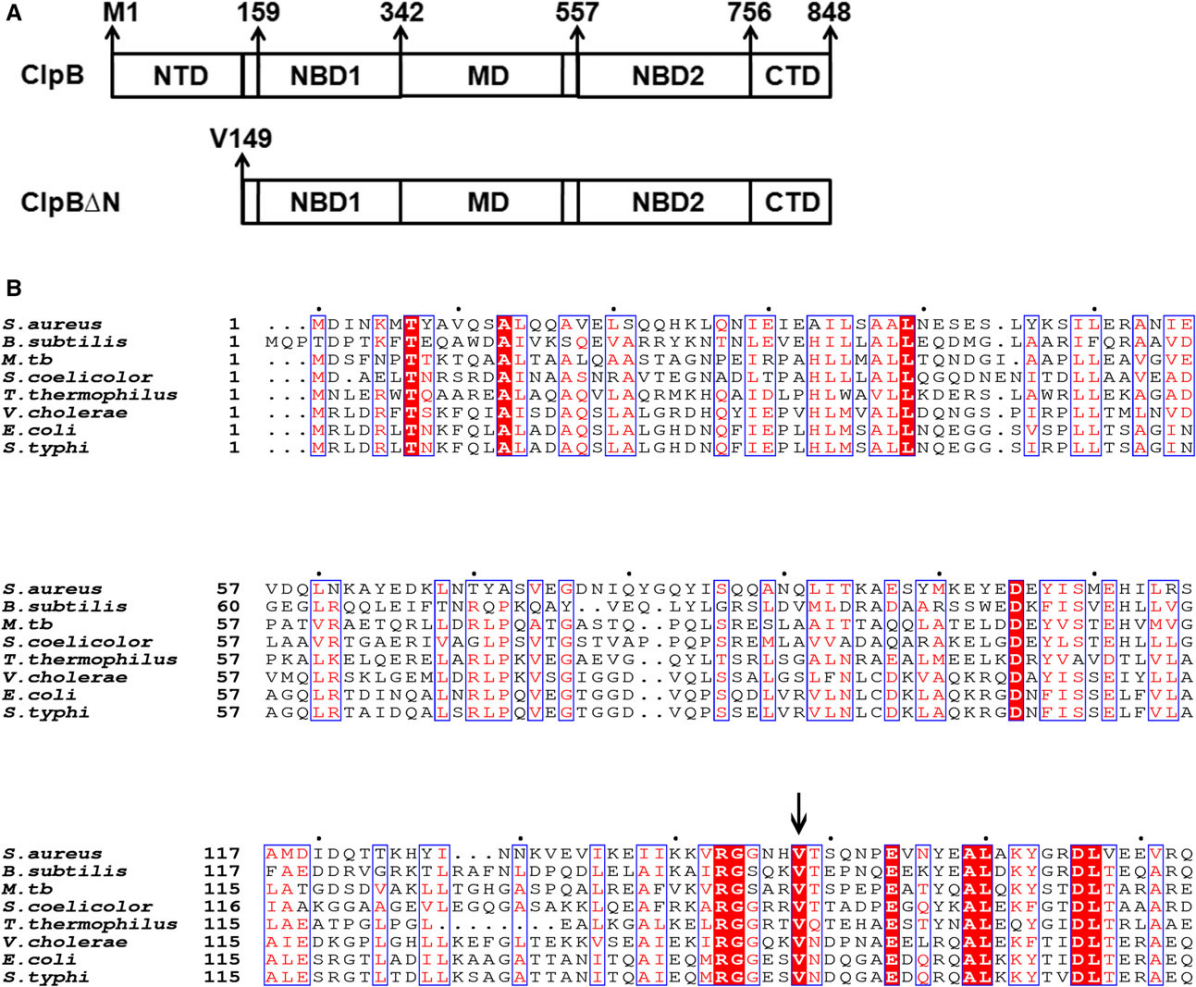
Domain notation of ClpB isoforms. (A) Diagrammatic representation of the postulated domain organization of full length ClpB protein and the N‐terminally truncated protein of *Mtb* based on similarity with the *E. coli* protein to show the NTD, NBD1, middle coiled coil domain (MD), NBD2 and C‐terminal domain (CTD), where residue numbers are given for the start of each domain. Val149 corresponds to the alternate translation start site that codes for ClpB∆N protein. (B) Multiple sequence alignment of the NTD of *Mtb* ClpB protein with that of different Gram positive and Gram negative human pathogens. Protein sequences were aligned using the clustalo and espript 3.0 online tool; arrow shows the conservation of internal translation site across the species. Characters in white on red background indicate completely conserved residues while those in red indicate similar residues.

The TDR Targets Database mentions the presence of a paralog of ClpB in the proteome of *Mtb*, which is indicative of a probable alternative form of the protein being produced. We show that, similar to *E. coli*, the *clpB* gene in *Mtb* expresses in two forms, a 93 kDa full length ClpB protein and an 80 kDa N‐terminally truncated protein. We have used the term ClpB∆N in this study to denote the N‐terminally truncated isoform. Sequence analysis of *Mtb* ClpB and ClpB∆N, using bioinformatic tools, reveals the presence of characteristic domains found in this family of proteins (Fig. 1A). A GTG codon at the 149th position, which acts as the non‐preferred start codon for ClpB∆N, was found to be highly conserved across multiple human pathogens (Fig. 1B). Except for *Mycoplasma* species, the two isoforms of ClpB have been found to be present in the majority of the organisms looked at 20. Nonetheless, there are conflicting reports about the role of the NTD in ClpB homologs. While it is dispensable for chaperonic activity and thermotolerance in *Thermus thermophilus* and *Synechococcus* sp. 21, 22, it was found to be involved in enhancing these activities in *E. coli* 18, 23, 24. Therefore, the importance of the conserved internal translation start site is still not clear.

Orthologs of ClpB are found in a wide variety of organisms, such as, bacteria, protozoans, yeast and plants. ClpB is known to confer thermotolerance on these organisms by virtue of its protein disaggregation activity 25, 26, 27, 28, 29. Intriguingly, ClpB is not found in animal genomes. ClpB has also been shown to regulate the distribution of irreversibly oxidized proteins amongst stressed mycobacterial cells 30. A recent study has also illustrated a functional similarity between the *Mtb* and *E. coli* ClpB proteins 31. Although the *Mtb* ClpB–KJE bi‐chaperone has been reconstituted *in vitro*, its functioning has not been fully explored 31. In this study, we have characterized the *Mtb* ClpB–KJE bi‐chaperone to delineate the mechanistic details of functioning of ClpB. Our study indicates that apart from reactivating the stress‐denatured proteins, ClpB could also be assisting in routine chaperoning of proteins. Further, we show that the reactivation activity of *Mtb* ClpB depends on the extent of aggregation of the substrate protein. In addition, different nucleotides render ClpB in different conformations, the consequences of which may be important for its activity.

In an attempt to understand the significance of conservation of the internal translation site in *Mtb clpB*, we have performed an in‐depth structure–function analysis of ClpB∆N protein and characterized the NTD by structure‐based mutagenesis. We report that the NTD is involved in substrate recognition and stabilizes the interaction of rather strongly aggregated proteins with the ClpB core. Our study recognizes some of the unacknowledged attributes of the NTD of *Mtb* ClpB and its unique connection to the activities of this protein.

## Materials and methods

### Plasmids

The DNA fragments coding for full length ClpB (residues 1–848) and N‐terminally truncated ClpB∆N (residues 149–848) were amplified by polymerase chain reaction using the genomic DNA of *Mtb* H_37_Rv as template and cloned between the BamHI and SalI sites of pQE30 expression vector to generate N‐terminally 6× His‐tagged construct. The gene encoding DnaJ1 was amplified as described above for ClpB and cloned between the NdeI and EcoRI sites of a T7 promoter‐based expression vector, pVEX11. The gene encoding GrpE was amplified as described above and cloned between the BamHI and HindIII sites in the pQE30 expression vector. The plasmid carrying the *dnaK* gene of *Mtb* was constructed as described previously 59. The primers listed in Table 1 were used to produce each of these gene fragments. All the plasmid constructs were verified by DNA sequencing.

**Table 1:** List of primers used in this study

Name	Region	Sequence (5′→3′)
JKB 1244	*Mtb* ClpB forward	ATTGACTTCAGGATCCGTGGACTCGTTTAACCCG
JKB 1245	*Mtb* ClpB reverse	TTCTATATAAGCTTTCAGCCCAGGATCAGCGA
JKB 1408	*Mtb* ClpB∆N forward	ATTACTTAGGATCCATGGTCGGGCTGGCCACC
JKB 1465	*Mtb* DnaK forward	AATTATGGATCCATGGCTCGTGCGGT
JKB 1487	*Mtb* DnaK reverse	AATTATAAGCTTTCACTTGGCCTCCCGGCCGTCGT
JKB 1467	*Mtb* GrpE forward	AATTATATGGATCCGTGACGGACGGAAAT
JKB 1468	*Mtb* GrpE reverse	AATTATATAAGCTTTTAACTGCCCGACGGTT
JKB 1469	*Mtb* DnaJ1 forward	AATTATATCATATGCATCATCACCATCACCACA
JKB 1470	*Mtb* DnaJ1 reverse	AATTATATGAATTCTCAGCGATTACCTGCCCAT
JKB 1623	ClpB L97D forward	CACCACCGCGCAGCAGGACGCCACCGAGCTGGACG
JKB 1624	ClpB L97D reverse	CGTCCAGCTCGGTGGCGTCCTGCTGCGCGGTGGTG
JKB 1625	ClpB L101Q forward	GCAGCTGGCCACCGAGCAGGACGACGAGTACGTCT
JKB 1626	ClpB L101Q reverse	AGACGTACTCGTCGTCCTGCTCGGTGGCCAGCTGC
JKB 1627	ClpB F140T forward	GGCGCTGCGGGAGGCGACCGTCAAGGTGCGCGGCA
JKB 1628	ClpB F140T reverse	TGCCGCGCACCTTGACGGTCGCCTCCCGCAGCGCC
JKB 1649	ClpB V14K forward	GCTGCGGGAGGCGTTCAAGAAGGTGCGCGGCAGCG
JKB 1650	ClpB V141K reverse	CGCTGCCGCGCACCTTCTTGAACGCCTCCCGCAGC

### Mutagenesis

Site‐directed mutagenesis by PCR was employed to obtain various N‐terminal point mutations in wild‐type (WT) ClpB protein of *Mtb*. For each of the mutations, two complementary 35‐mer DNA primers, carrying the desired mutation in the middle of the sequence, were designed. The plasmid containing WT *clpB* DNA was used as the template to produce a mutant *clpB* DNA fragment, using the two primers and *Pfu* polymerase. The amplified product was digested thereafter with 10 U of *DpnI* for 3 h at 37 °C. Subsequently, competent *E. coli* XL1‐blue cells were transformed with this mixture and plated on Luria Bertani (LB)–ampicillin plates to obtain discrete colonies. The clones were confirmed by DNA sequencing to have the desired mutation.

### Protein expression and purification

Full length ClpB, its truncated isoform ClpB∆N and the mutants were overexpressed in *E. coli* strain M15 and purified as described earlier 40 with slight modifications. Briefly, transformed *E. coli* cells were grown at 37 °C to A_600_ ~ 0.8 in LB broth containing 100 μg·mL^−1^ ampicillin and induced with 1 mm isopropyl‐β‐d‐thiogalactoside (IPTG) for 4 h. Cells were harvested by centrifugation and resuspended in buffer A (50 mm Tris/HCl, pH 7.5, 100 mm KCl, 10% (v/v) glycerol, 1 mm EDTA and 1 mm DTT) to disrupt cells by sonication. The lysate was centrifuged at 20 000 ***g*** for 45 min at 4 °C. Polyethyleneimine was added slowly to the supernatant to a final concentration of 0.04% (w/v) and centrifuged again at 4 °C for 45 min. The supernatant was further subjected to ultracentrifugation at 100 000 ***g*** for 1 h at 4 °C. The supernatant was then loaded onto a Ni–nitrilotriacetic acid column pre‐equilibrated with buffer A. Subsequently, the column was washed with buffer A containing 25 mm imidazole and proteins were eluted with buffer A containing 300 mm imidazole. Collected fractions were analyzed on SDS/PAGE, and fractions containing the highest amount of protein were pooled and precipitated with 60% ammonium sulfate. The precipitate was collected by centrifugation and resuspended in buffer A. The sample was then loaded onto a 1 × 60 cm Superdex‐200 column and eluted with buffer A. The fractions were analyzed by SDS/PAGE, and those containing proteins of the highest purity were pooled and concentrated with ammonium sulfate. The samples were dialyzed extensively in buffer B containing 50 mm Tris/HCl, pH 7.5, 20 mm MgCl_2_, 200 mm KCl, 10% (v/v) glycerol, 1 mm EDTA and 1 mm DTT.

The co‐chaperone DnaK was purified in the same manner as described previously 59. For purifying DnaJ1, the plasmid encoding DnaJ1 was overexpressed in *E. coli* strain BL21. The cells were grown in LB broth at 37 °C to *D*_600_ ~ 0.8 and induced with 1 mm IPTG for 3 h. The protein was purified from the soluble fraction as described earlier 60. Briefly, cells were washed once with ice cold STE buffer (10 mm Tris/HCl, pH 8.0, 150 mm NaCl and 1 mm EDTA), resuspended in STE buffer containing lysozyme to a final concentration of 100 μg·mL^−1^ and incubated on ice for 15 min. Subsequently, 5 mm DTT, 1.5% (w/v) sarcosine detergent and 1 mm CaCl_2_ were added and cells were disrupted by sonication. The lysate was centrifuged for 10 min at 16 000 ***g*** and 4 °C. Triton X‐100 was added to the supernatant to a final concentration of 4% (v/v) and ultracentrifugation was carried out at 4 °C for 45 min at 100 000 ***g***. The supernatant was dialyzed against buffer C (10 mm Tris/HCl, pH 8.0, 150 mm NaCl, 1 mm CaCl_2_, 0.5% (w/v) sarcosine and 1% (v/v) Triton X‐100) and loaded onto a Ni–nitrilotriacetic acid column pre‐equilibrated with buffer D (10 mm Tris/HCl, pH 8.0, 150 mm NaCl, 0.25% (w/v) sarcosine, 0.5% (v/v) Triton X‐100 and 10% (v/v) glycerol). The column was washed with buffer D containing 25 mm imidazole and protein was eluted with buffer D containing 250 mm imidazole. The eluates were analyzed by SDS/PAGE and fractions containing the highest amount of protein were pooled and dialyzed against buffer E (10 mm Tris/HCl, pH 8.0, 150 mm NaCl, 0.1% (w/v) sarcosine, 0.25% (v/v) Triton X‐100 and 10% (v/v) glycerol). The GrpE‐encoding plasmid was overexpressed in *E. coli* strain M15 in LB broth at 37 °C to *D*_600_ ~ 0.8 and induced with 1 mm IPTG for 4 h. The protein was purified under native conditions as described earlier for DnaK 59.

### Gel filtration chromatography

Preparative as well as analytical gel filtration chromatography was performed using a 1 × 60 cm Superdex‐200 column (GE Healthcare, Chicago, IL, USA) equilibrated with buffer A. The column was run at a constant flow rate of 0.4 mL·min^−1^ using a GE ÄKTA Purifier chromatography system (GE Healthcare) with a diode array UV‐visible absorption detector. If the experiment involved use of nucleotides, the proteins were incubated with 5 mm respective nucleotide for 10 min at room temperature before loading onto the column. Elution of the proteins was carried out with buffer A. High molecular mass protein standards obtained from GE Healthcare were eluted in same buffer according to the manufacturer's protocol.

### Circular dichroism spectroscopy

The CD spectra of 1 μm protein solution in buffer A were recorded in the far‐UV range (200–250 nm) at 25 °C with a JASCO J‐815 spectropolarimeter using a cell of 0.1 cm optical path. The measurement was done at a scan speed of 100 nm·min^−1^ with a sensitivity of 50 mdeg and a response time of 1 s. The spectra were averaged over five scans. The results are presented as mean residue ellipticity. To study thermal stability in terms of *T*_m_, i.e. the mid‐point of thermal denaturation, heat‐induced denaturation of the proteins was monitored with the spectropolarimeter while the temperature of the cell was increased gradually at a rate of 1 °C·min^−1^ using a Peltier‐type temperature controller. Change in [θ]_222_ was monitored in the temperature range 20–90 °C. Assuming that heat induces a two‐state transition, i.e. native state (*N*) to denatured state (*D*), each denaturation curve was analyzed by fitting in Equation (1) to obtain the value of *T*_m_ where, *y*(*T*) is the optical property at temperature *T*(K), *yN*(*T*) and *yD*(*T*) are the optical properties of the native and denatured states of the protein at temperature *T* (K) and *R* is the molar gas constant.

(1) $$y(T)=\frac{yN+yD\text{exp}\left[-\frac{\Delta H_{m}}{R(\frac{1}{T}-\frac{1}{T_{m}})}\right]}{1+\text{exp}\left[-\frac{\Delta H_{m}}{R(\frac{1}{T}-\frac{1}{T_{m}})}\right]}$$

### Biochemical assays

#### ATPase activity assay

Proteins were incubated at 37 °C for 30 min in the ATPase assay buffer (50 mm Tris/HCl, pH 7.5, 150 mm KCl, 20 mm MgCl_2_, 1 mm DTT and 10% (v/v) glycerol) with 5 mm ATP containing [^32^γP]ATP, and indicated amounts of ADP, if mentioned. The reaction was stopped by adding 50 μL of chilled activated charcoal (100 mg·mL^−1^ in 1 m HCl). The mixture was then incubated on ice for 15 min with intermittent vortexing, and centrifuged twice for 30 min at 16 000 ***g*** and 4 °C. Radioactivity in the supernatant was measured using a liquid scintillation counter and the concentration of released P_i_ was calculated in terms of specific activity of the protein.

#### Prevention of aggregation of luciferase

Heat induced aggregation of 300 nm recombinant firefly luciferase (Sigma‐Aldrich, St Louis, MO, USA) in buffer G (50 mm HEPES/KOH, pH 7.5, 100 mm KCl, 10 mm MgCl_2_, 1 mm EDTA, 1 mm DTT and 10% (v/v) glycerol) was monitored by UV absorbance at 320 nm for 25 min at 43 °C in a Varian Cary Eclipse fluorescence spectrophotometer (Santa Clara, CA, USA) equipped with a temperature controller. Various concentrations of ClpB or ClpB∆N protein were added to the reaction, with 5 mm of ATP or other indicated nucleotides, prior to heat aggregation and their effect on the aggregation of luciferase was recorded. The end‐point value of the absorbance of aggregated luciferase alone was considered as 100% aggregation. The percentage aggregation of other variables was calculated relative to this value.

#### Reactivation of heat‐aggregated luciferase

Luciferase (100 nm), diluted in buffer G, was heat aggregated at 43 °C in a water bath for 5 or 15 min to generate small or large aggregates, respectively. Aggregates were diluted in reactivation buffer (50 mm triethanolamine/HCl, pH 7.8, 100 mm KCl, 20 mm Mg (OAc)_2_, 1 mm β‐mercaptoethanol, 10% (v/v) glycerol and 1 mm EDTA) to a final concentration of 100 nm in the presence of 5 mm ATP and 2 μm ClpB or its variants, with or without 1 μm DnaK, 0.5 μm DnaJ1 and 0.2 μm GrpE (KJE). The reaction mixture was then incubated at 25 °C for 45 min. The reactivation of aggregated luciferase was assessed in terms of the luciferase activity by measuring luminescence in a Berthold MicroLumat luminometer. For this, 5 μL of reactivation reaction mixture was diluted 14‐fold in a buffer containing 50 mm Tris/HCl, pH 7.8, 2.5 mm MgSO_4_, 1 mm MgCl_2_, 0.1 mm EDTA, 15 mm DTT, 1 mm ATP and 270 μm coenzyme A. The substrate, d‐luciferin, was added to a final concentration of 500 μm and luminescence was recorded.

#### Reactivation of heat‐aggregated malate dehydrogenase

Malate dehydrogenase (MDH) from pig heart (Sigma‐Aldrich) was diluted to 2 μm in denaturation buffer (8 m urea, 40 mm DTT and 10% (v/v) glycerol) and incubated at 47 °C for 15 min. The mixture was further diluted to 1 μm in reactivation buffer, mixed vigorously and again incubated at 47 °C for 15 or 30 min, in order to produce small or large aggregates of MDH, respectively, as described elsewhere 40. Aggregation was stopped by keeping samples on ice for 5 min. Subsequently, an aliquot was taken from this mixture, diluted 8‐fold in reactivation buffer containing 5 mm ATP and 2 μm ClpB or its variants, with or without 1 μm DnaK, 0.5 μm DnaJ1 and 0.2 μm GrpE, and incubated at 30 °C for 60 min. The disaggregation of aggregated MDH was measured as a function of its enzymatic activity for which it consumes NADH in order to convert oxaloacetic acid into malic acid. Aliquots were withdrawn from the reactivation reaction mixture, at the end of the incubation, and diluted 20‐fold into the assay buffer (50 mm Tris/HCl, pH 7.8, 5 mm MgCl_2_, 1 mm DTT, 0.3 mm oxaloacetate and 0.15 mm NADH) and incubated at 30 °C. Absorption at 340 nm was measured after 10 min. The enzymatic activity of native MDH was set as 100%.

### Dynamic light scattering to estimate aggregate size

Dynamic light scattering measurements were carried out with an RiNA Laser Spectroscatter‐201 to obtain hydrodynamic radii of small and large aggregates of MDH and luciferase at 25 °C. Protein aggregates (400 μL) were prepared in appropriate buffers filtered through a 0.22 μm filter and used for this study. Measurements were made at a fixed angle of 90° using an incident laser beam of 689 nm. Ten measurements were made with an acquisition time of 20 s for each sample at a sensitivity of 10%. The data were analyzed using the pmgr v3.01 p17 software program provided by the manufacturer to obtain the hydrodynamic radius of the protein aggregates. The average hydrodynamic radii of different aggregates were plotted using prism (GraphPad Software Inc., San Diego, CA, USA).

### Fluorescence polarization substrate binding assay

FITC‐labelled casein was purchased from Sigma‐Aldrich and used as the substrate for fluorescence polarization experiments. To determine the substrate binding affinity of WT ClpB protein and its variants, different dilutions of each protein were incubated for 20 min with 70 nm FITC–casein in reactivation buffer containing 2 mm ATPγS. Binding was detected by following the fluorescence polarization signal, measured in a Horiba Scientific (Kyoto, Japan) fluorescence sprectrometer. Each sample was read at least 10 times to get an average reading and the data were fitted to a one‐site binding equation using prism to deduce dissociation constants (*K*_d_).

### Expression analysis in *Mtb* H37Rv under heat shock

To investigate the expression profile of full length ClpB and ClpB∆N, *Mtb* strain H37Rv was cultured in 7H9 Middlebrook medium containing 0.5% (v/v) glycerol, 0.05% (v/v) Tween 80 and supplemented with 10% (v/v) albumin, dextrose and catalase at 37 °C and 100 r.p.m. The cultures were shifted to 42 °C or continued at 37 °C once they reached *D*_600_ = 0.2, and maintained at the respective temperature for the next 5 days. Aliquots were collected every 24 h for preparing whole cell lysates that were further quantified by Bradford reagent. Equal amount of lysates were then resolved on 10% SDS/PAGE, transferred onto poly(vinylidene difluoride) membrane, and probed with polyclonal anti‐ClpB antibody raised in rabbit.

### Statistical analysis

The standard errors of the mean (SEM) were calculated using the prism software. Student's *t* test (unpaired, two‐tailed) was used to analyze the significance of results that were compared. Values of *P* < 0.05 were considered as statistically significant.

## Results

### Expression profile of ClpB isoforms under heat stress

**Figure 2:**
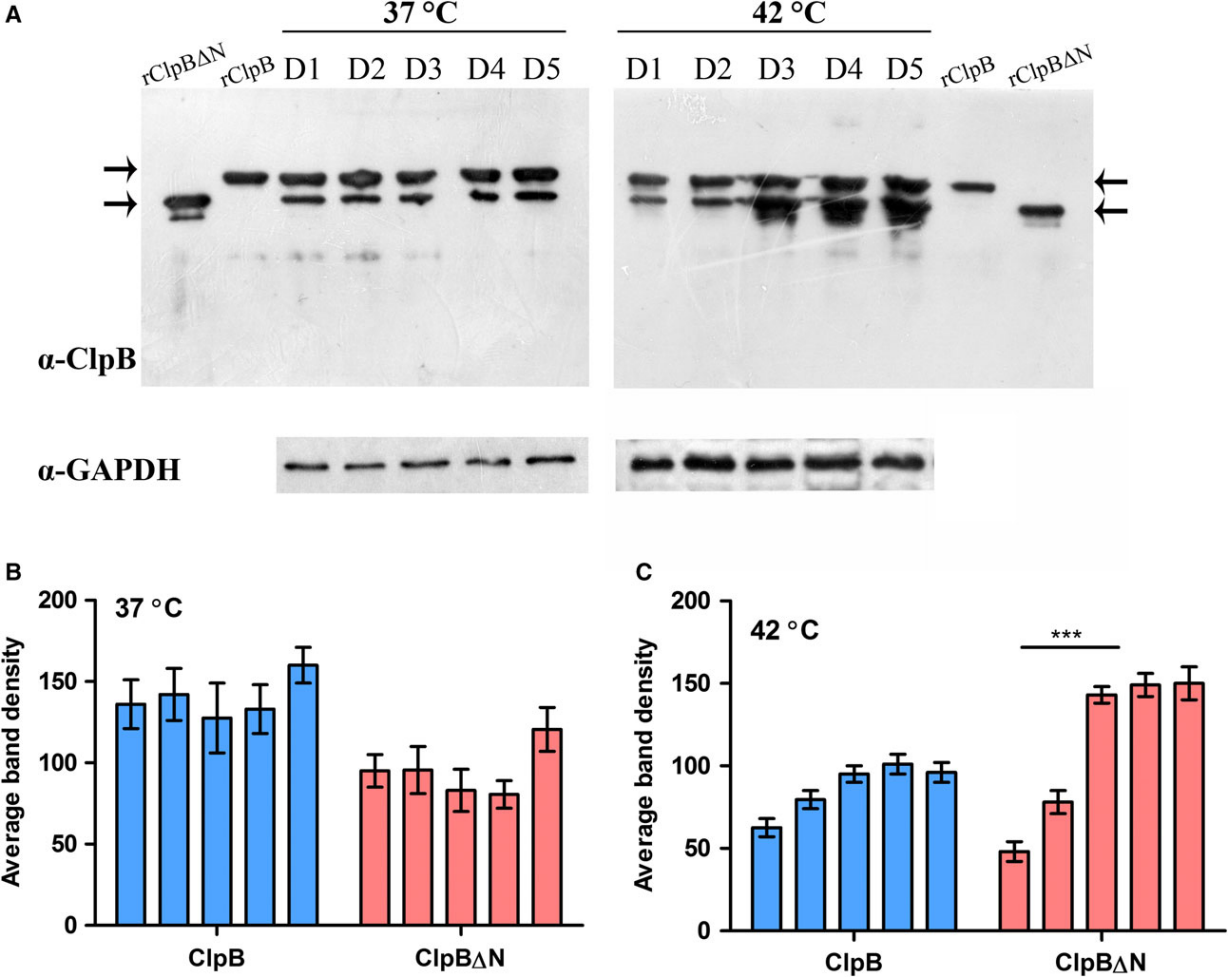
Expression profile of ClpB *versus* ClpB∆N under normal and heat shock conditions. (A) Western blot analysis of the whole cell lysates of *Mtb* H_37_Rv cultures, grown under normal or heat shock conditions was performed with polyclonal α‐ClpB antibody at 1 : 25 000 dilution. Cultures were grown at 37 and 42 °C simultaneously for 5 days to mimic normal and heat shock growth conditions, respectively; see ‘Materials and methods’ for details. Lanes 1–5 in the left and right panels represent cell lysate of each day. A similar blot shows the position of recombinant ClpB and ClpB∆N (placed in the middle, labelled as ClpB and ClpB∆N) proteins on the blot. *Mtb* glyceraldehyde 3‐phosphate dehydrogenase (GAPDH) has been used as the loading control. (B,C) Band intensity of the blot for 37 and 42 °C, respectively, was measured using imagej software. Statistical analysis was performed using two‐way anova and the error bars represent the SEM.

To confirm the presence of the N‐terminally truncated form of ClpB intracellularly, we probed the whole cell lysate of the *Mtb* culture grown at 37 °C with polyclonal anti‐ClpB antibody and compared the bands with recombinant full length ClpB and ClpB∆N protein bands (Fig. 2A, left panel). We found bands corresponding to the recombinant proteins, ClpB and ClpB∆N, in the whole cell lysate, confirming their presence *in vivo*. ClpB in other organisms has been shown to be up‐regulated under thermal stress. It has been shown in *Mtb* also that the *clpB* gene, although non‐essential under normal growth conditions, becomes indispensable when the bacilli encounter stress conditions 30. However, the regulation of ClpB *vs*. ClpB∆N under stress conditions has not been studied. We investigated the expression levels of the two isoforms of ClpB with respect to each other, under normal growth condition *versus* heat stress condition. Figure 2A shows that both the forms of ClpB are expressed at 37 °C as well as 42 °C. While at 37 °C both the isoforms were expressed at a constant level for 5 days, the levels of ClpB protein were significantly higher than that of ClpB∆N, as analyzed by band densitometry (Fig. 2B). Under heat shock conditions, the expression of both ClpB and truncated ClpB∆N was affected and varied from day 1 to day 5. ClpB∆N protein expressed at a constant level for the initial 2 days but showed an increase from day 3 onwards (Fig. 2A,C), whereas ClpB showed a non‐significant increase in expression towards the end of the experiment. In addition, a significant difference in the pattern of ClpB *vs*. ClpB∆N levels at 37 °C from that at 42 °C was observed (Fig. 2 B,C). Such a differential expression of ClpB isoforms suggests that there could be differences in the structural and/or biochemical properties of the two isoforms enabling them to function differently under stress.

### ClpB and ClpB∆N have comparable secondary structures and are thermodynamically stable

**Figure 3:**
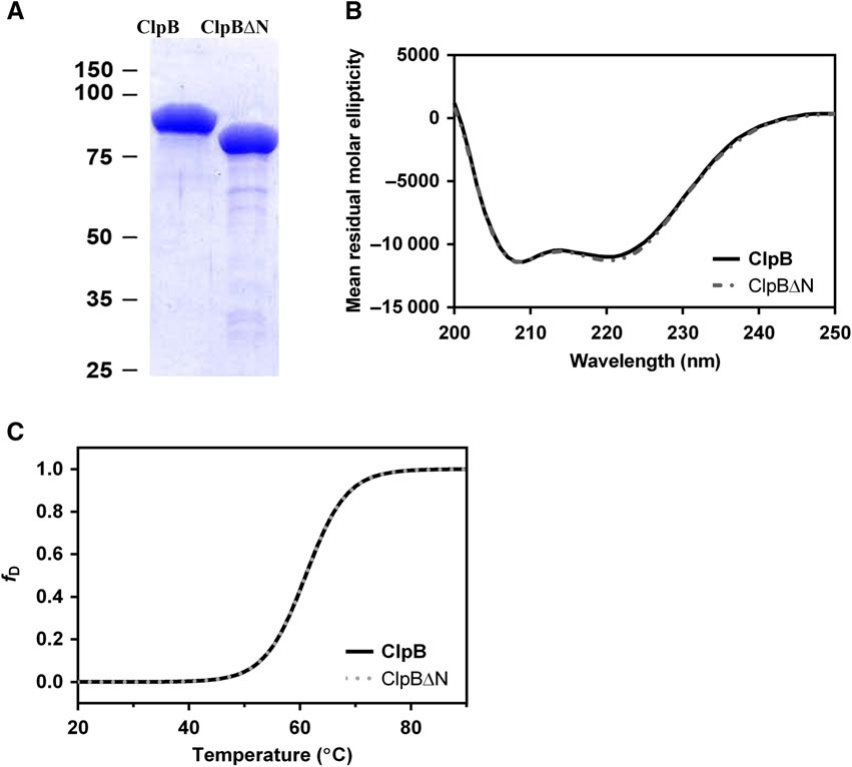
Characterization of structural properties of ClpB and ClpB∆N proteins. (A) 12.5% SDS/PAGE showing purified recombinant ClpB and ClpB∆N proteins used in this study; numbers on the left indicate molecular masses in kDa. (B) CD spectra of 1 μm ClpB and ClpB∆N proteins in the far‐UV region (200–250 nm), represented as mean residual molar ellipticity. The spectra were measured at 25 °C in buffer C. (C) Thermal denaturation of ClpB and ClpB∆N proteins recorded using CD spectroscopy at temperatures ranging from 20 to 90 °C. Results are expressed in the form of decrease in helicity as a function of temperature (*f*_D_).

We cloned the DNA encoding *Mtb* ClpB full length and *Mtb* ClpB lacking 149 N‐terminal amino acids termed ClpB∆N individually, expressed them in *E. coli* and purified them to near homogeneity (Fig. 3A). To evaluate whether the lack of the NTD in ClpB∆N leads to any change in the secondary structure of the protein, we recorded the CD spectra of full length ClpB and the ClpB∆N variant (Fig. 3B). The CD spectra of both the proteins were found to be similar, and showed minima at 208 and 222 nm, a pattern typical of proteins abundant in α‐helical content. The CD spectral analysis indicated that despite losing the NTD, ClpB∆N was able to maintain the overall secondary structure of the full length protein. Further, to check if the deletion had affected the thermodynamic stability of ClpB∆N, we measured CD spectra of the two isoforms at various temperatures. A decrease in helicity of both the proteins as a function of temperature was seen only beyond 45 °C (Fig. 3C). Both ClpB and ClpB∆N transitioned cooperatively under thermal denaturation with a *T*_m_ of 59.7 and 59.5 °C, respectively, indicating that N‐terminal deletion does not destabilize the conformation of ClpB∆N.

### ClpB∆N self‐associates in a concentration‐ and nucleotide‐dependent manner similar to ClpB

**Figure 4:**
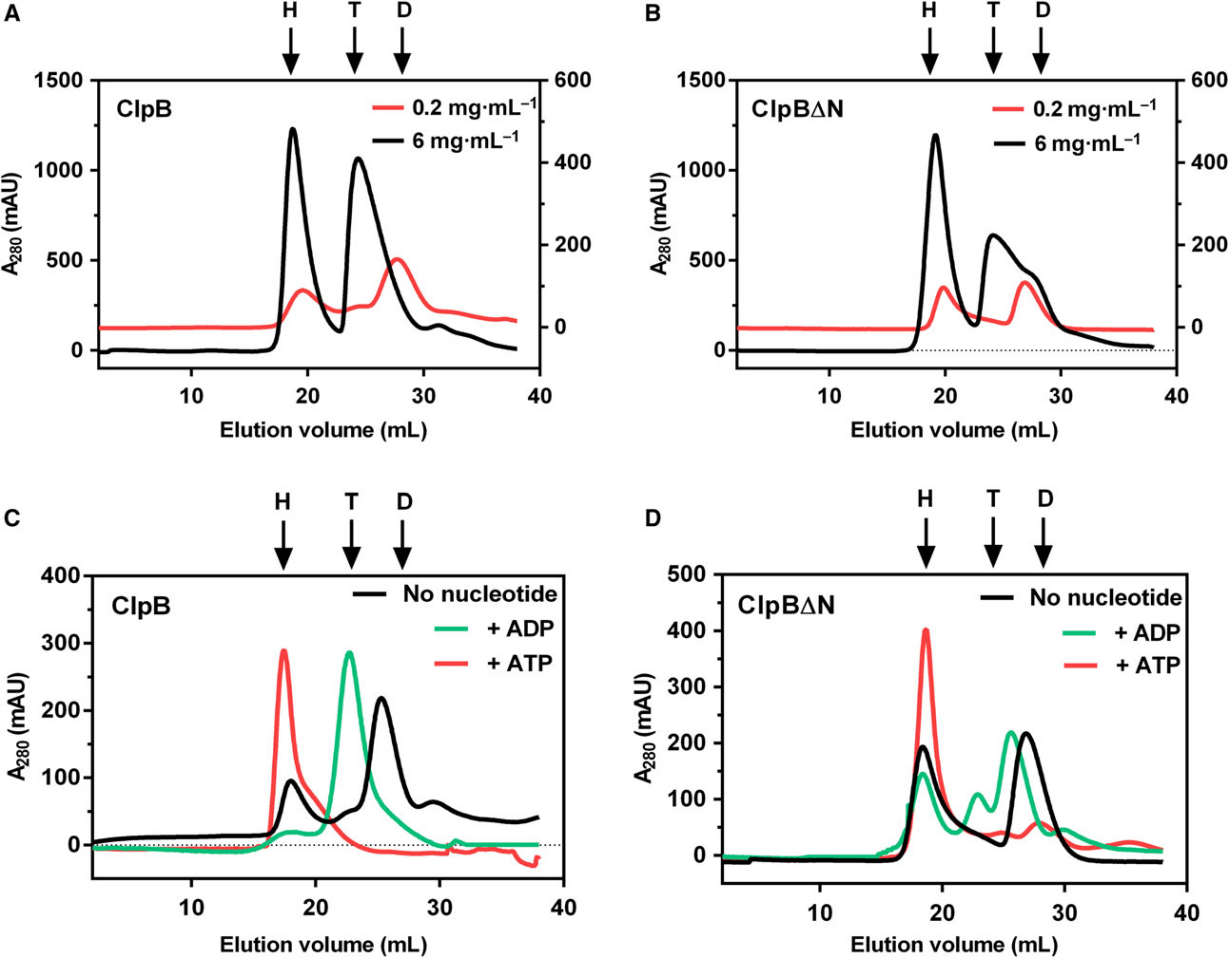
Analysis of the oligomeric status of ClpB isoforms. The oligomeric status of ClpB∆N protein analyzed under different conditions by gel filtration chromatography and compared with that of the full length protein. ClpB (A) and ClpB∆N (B) proteins were loaded on to Superdex‐200 column at a concentration of 0.2 mg·mL^−1^ (right *y* axis) or 6 mg·mL^−1^ (left *y* axis) and the effect of concentration on their self‐association was analyzed. (C,D) Effect of different nucleotides on the oligomerization of ClpB (C) and ClpB∆N (D). Proteins (0.2 mg·mL^−1^) were incubated with 5 mm ATP or ADP for 10 min before loading onto the column. Arrows mark the position of elution of hexamer (H), tetramer (T) or dimer (D) that corresponds to the elution volume of molecular mass standards: thyroglobulin (660 kDa), ferritin (440 kDa) and aldolase (168 kDa). Panels are representative of experiments done at least twice with different protein preparations.

To investigate the oligomeric status of the N‐terminally truncated isoform of ClpB vis‐à‐vis full length ClpB, we analyzed the two proteins by gel filtration chromatography at two different concentrations (Fig. 4A,B). The monomeric molecular mass of *Mtb* ClpB is 93 kDa and that of ClpB∆N is 80 kDa, whereas their calculated hexameric molecular masses are ~ 560 and 480 kDa, respectively. Thyroglobulin (*M* 660 kDa), ferritin (*M* 440 kDa) and aldolase (*M* 168 kDa), corresponding to the calculated molecular mass of hexameric, tetrameric and dimeric forms of ClpB protein, respectively, were used as molecular mass standards. At a concentration of 0.2 mg·mL^−1^, both the isoforms of ClpB existed as hexamers and dimers (Fig. 4A,B). In contrast, increasing the concentration to 6 mg·mL^−1^ resulted in a shift of dimer peaks towards higher order oligomers suggesting that these proteins exhibit a concentration‐dependent self‐association in solution (Fig. 4A,B). At higher concentration, major fractions of both the proteins were found as hexamers and tetramers. The population of hexameric species was found to be similar in both ClpB and ClpB∆N, at low as well as at high concentrations, which indicated that the loss of the NTD did not affect the propensity of the protein to hexamerize.

It is well established that the AAA^+^ proteins work in close association with nucleotides 3, 32. In light of this, we studied the effect of different nucleotides on the oligomerization of ClpB and ClpB∆N. Proteins were incubated with ATP or ADP and then analyzed by gel filtration chromatography. A complete shift towards the hexameric form was observed for both the proteins in the presence of ATP (Fig. 4C,D). Addition of ADP, however, resulted in a complete shift towards the tetrameric form for full length ClpB, while ClpB∆N showed a mixed distribution of oligomers. Such a switch in the oligomeric status of ClpB and ClpB∆N indicates that the stable hexamerization of these proteins is dependent on ATP.

### ClpB∆N has enhanced ATP hydrolyzing activity but is weakly modulated by DnaK or substrates

**Figure 5:**
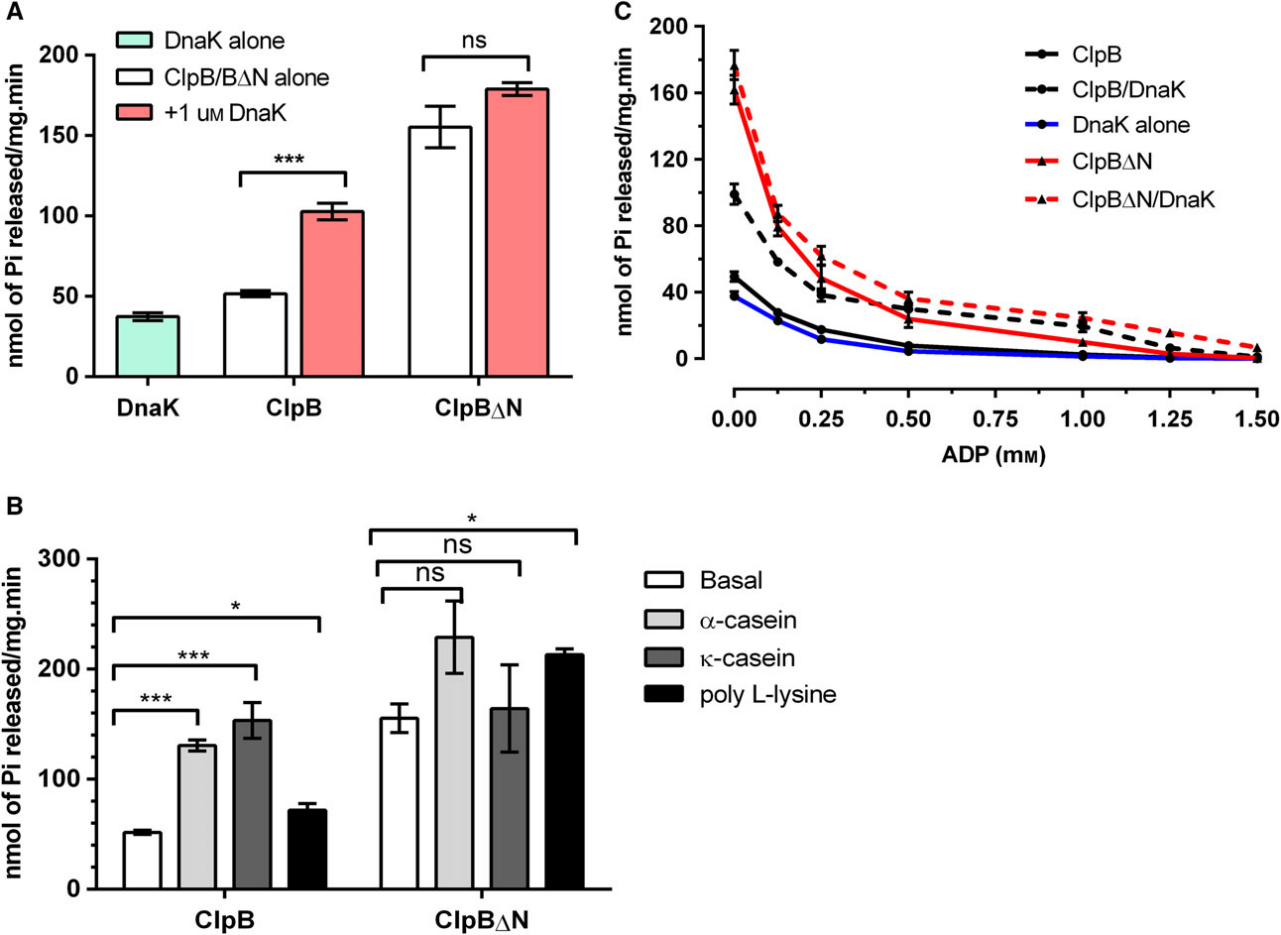
Effect of N‐terminal deletion on ATP hydrolysis by ClpB. (A) Comparison of specific ATPase activity of ClpB∆N with full length ClpB. Modulation of specific ATPase activity by addition of 1 μm DnaK to ClpB and ClpB∆N proteins is also shown. ***P <* *0.001. (B) Modulation of specific ATPase activity by addition of different disordered substrates: α‐casein (0.2 mg·mL^−1^), κ‐casein (0.2 mg·mL^−1^), and poly‐l‐lysine (0.04 mg·mL^−1^). *P <* *0.05, ***P ≤* *0.0001. (C) Effect of ADP on the specific ATPase activity of ClpB and ClpB∆N in the presence or absence of 1 μm DnaK. The data represent mean ± SEM for three independent experiments done in triplicate. Statistical analysis was performed using Student's *t* test.

We further evaluated the effect of loss of the NTD on its ATP binding and hydrolysis function. The ATPase activity of full length ClpB was 52 ± 1.8 nmol ATP per mg protein per minute, whereas ClpB∆N showed a 3‐fold higher activity (Fig. 5A). ClpB has been shown to act in close association with DnaK protein in other organisms 33, 34 as well as in *Mtb* 31. We expressed and purified *Mtb* DnaK and characterized its ATP hydrolysis function (Fig. S1A,B). It was found to have slightly lower basal ATPase activity compared to that of ClpB, possibly because it has only one NBD. *Mtb*, contrary to many pathogenic protozoans, is found to have two DnaJ proteins, J1 and J2 35, both of which are capable of interacting with and stimulating DnaK. At least one DnaJ has been reported to be dispensable 31. In this study, we recombinantly produced *Mtb* DnaJ1 for our further experiments and purified and tested its activity. The DnaJ1 protein was found to stimulate the ATPase activity of DnaK in a dose‐dependent manner (Fig. S1B), which corroborates the recent study in *Mtb* by Lupoli *et al*. 31.

We further evaluated the effect of DnaK interaction with ClpB and ClpB∆N on their ATPase activity to analyze if the N‐terminally truncated protein behaved differently in terms of interaction or activity. As shown in Fig. 5A, the ATP hydrolysis activity of ClpB was enhanced 2‐fold in the presence of 1 μm DnaK. ClpB∆N, on the other hand, showed an insignificant increase in the ATPase activity in the presence of DnaK (Fig. 5A). To test whether these proteins could also be modulated by their substrates, we measured their ATPase activity in the presence of three intrinsically unstructured proteins, namely, α‐casein, κ‐casein and poly‐l‐lysine with molecular masses of 15–30 kDa. Poly‐l‐lysine may not necessarily act as a substrate of ClpB, but it is a known activator of ClpB ATPase activity 36. As is well known for its homologs 21, 36, *Mtb* ClpB was also found to be stimulated by these unstructured proteins (Fig. 5B). Interestingly, all three proteins modulated the ATPase activity of ClpB differently, with κ‐casein stimulation being the highest, which was more than 3‐fold higher (Fig. 5B). This analysis indicated that ClpB may possess a substrate differentiation property that possibly depends on the extent of disorder of the substrate. However, ClpB∆N seemed to have lost this ability of modulation as the substrates used could not stimulate its ATPase activity at all (Fig. 5B). Thus, it appears that the ability to stably bind and distinguish between substrates comes from the NTD of the protein.

*Mtb* ClpB and ClpB∆N prove to be highly efficient ATP hydrolyzing machines *in vitro*. However, the cellular environment has different levels of adenine nucleotides at different stages. The average intracellular ATP concentration in the *E. coli* cells is reported to be around 1–5 mm depending on the growth conditions while that of ADP remains ~ 2–3‐fold lower 37, 38. In the absence of any relevant report on the adenine pool of mycobacteria, we assumed the ratio of ATP to ADP to be similar to that in *E. coli*. We were interested to see the effect of different concentrations of ADP on the ATPase activity of ClpB and ClpB∆N under saturating concentrations of ATP. We observed that even under saturating levels of ATP (5 mm here), ClpB lost more than 80% of its ATPase activity upon addition of as low a concentration as 0.25 mm ADP in the reaction while ClpB∆N lost about 75% of its ATPase activity under the same condition (Fig. 5C), indicating that they have significantly higher affinity for ADP as compared to ATP. Complete loss of ClpB ATPase activity was seen at 0.5 mm ADP, whereas double the concentration was required to completely inhibit ClpB∆N (Fig. 5C). However, the inhibition could be restricted or partially limited upon addition of DnaK, as ClpB and ClpB∆N showed 40% and 17% ATPase activity even at 0.5 and 1 mm ADP, respectively, in the presence of DnaK (Fig. 5C). A similar observation has been reported with the yeast ClpB homolog, Hsp104 39.

### ClpB isoforms can act as stand‐alone chaperones and prevent aggregation of luciferase

**Figure 6:**
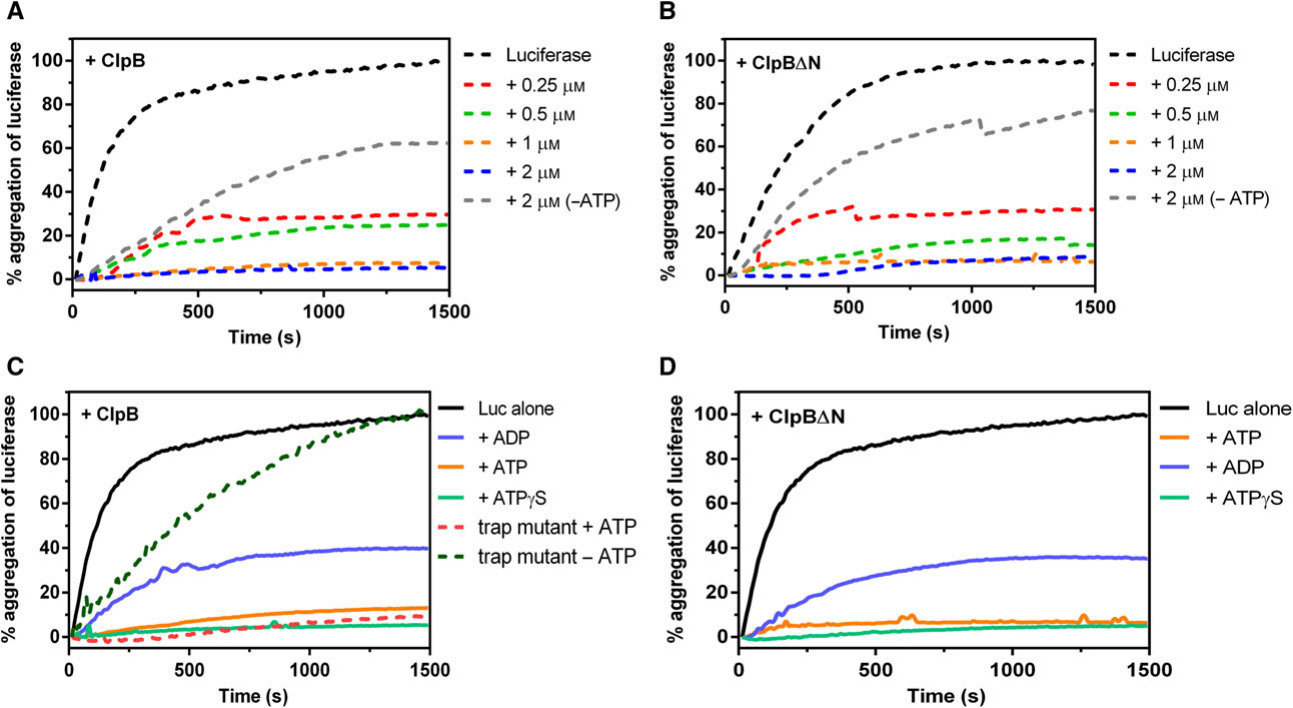
Prevention of aggregation activity of ClpB and ClpB∆N. (A,B) Prevention of aggregation activity of ClpB or ClpB∆N protein was followed kinetically in a dose‐dependent manner; 0.3 μm luciferase was heat‐aggregated in the presence/absence of ClpB (A) or ClpB∆N (B) protein and change in turbidity of the reaction mixture was measured at 320 nm; 5 mm ATP was been added to every reaction unless otherwise mentioned. (C,D) Effect of different nucleotides on the prevention of aggregation activity of ClpB (C) and ClpB∆N (D) proteins, respectively; 5 mm of each nucleotide was added where mentioned. Absorbance of aggregated luciferase at 1500 s has been considered as 100%. All other absorbance values are relative to this. Trap mutant of ClpB was used to confirm the ATP hydrolysis independence (C). Concentration of ClpB and its variants was kept at 1 μm. Figures are representative of experiments done at least thrice with different substrate and protein preparations.

For the mechanistic analysis of protein disaggregation by ClpB or ClpB∆N and the KJE bi‐chaperone system, we analyzed the aggregation kinetics of luciferase in the presence of these proteins at 43 °C (Fig. 6A,B). In the presence of ATP, ClpB prevented the aggregation of luciferase in a concentration‐dependent manner, and as low as 0.25 μm ClpB could maintain about 70% of the luciferase in a soluble state (Fig. 6A). Upon increasing the concentration by 8‐fold, ClpB was able to ‘hold’ about 95% of the luciferase in an unaggregated state. The prevention of aggregation activity of ClpB∆N was found to be comparable to that of ClpB at all concentrations, indicating that the loss of the NTD had not hampered the holdase activity of ClpB∆N (Fig. 6B). However, in the absence of ATP, prevention of aggregation activity dropped to around 38% and 24% for ClpB and ClpB∆N, respectively, with the highest concentration of proteins (Fig. 6A,B) indicating that a stable interaction with the substrate requires the ATP‐bound state of the hexamers.

Further, we investigated the effect of different nucleotides on the prevention of aggregation activity of ClpB and ClpB∆N. At 5 mm ATP or ATPγS, complete prevention of aggregation was observed with ClpB, whereas the addition of 5 mm ADP to ClpB resulted in only 60% prevention (Fig. 6C). We observed a similar pattern in the case of ClpB∆N as well (Fig. 6D). This observation indicated that for prevention of aggregation ATP hydrolysis is not required. To validate the finding that ClpB isoforms could prevent aggregation in an ATP‐hydrolysis‐independent manner, we constructed a trap mutant of ClpB, D278N/D679N, which is defective in both the Walker B motifs and allows binding but not hydrolysis of nucleotides. Figure 6C shows that the trap mutant was as efficient as the WT ClpB in preventing aggregation of luciferase in the presence of ATP, further confirming that it is not the ATP hydrolysis but binding that influences the prevention of aggregation activity of the ClpB isoforms.

### The NTD regulates the reactivation activity of *Mtb* ClpB by facilitating stable substrate binding

**Figure 7:**
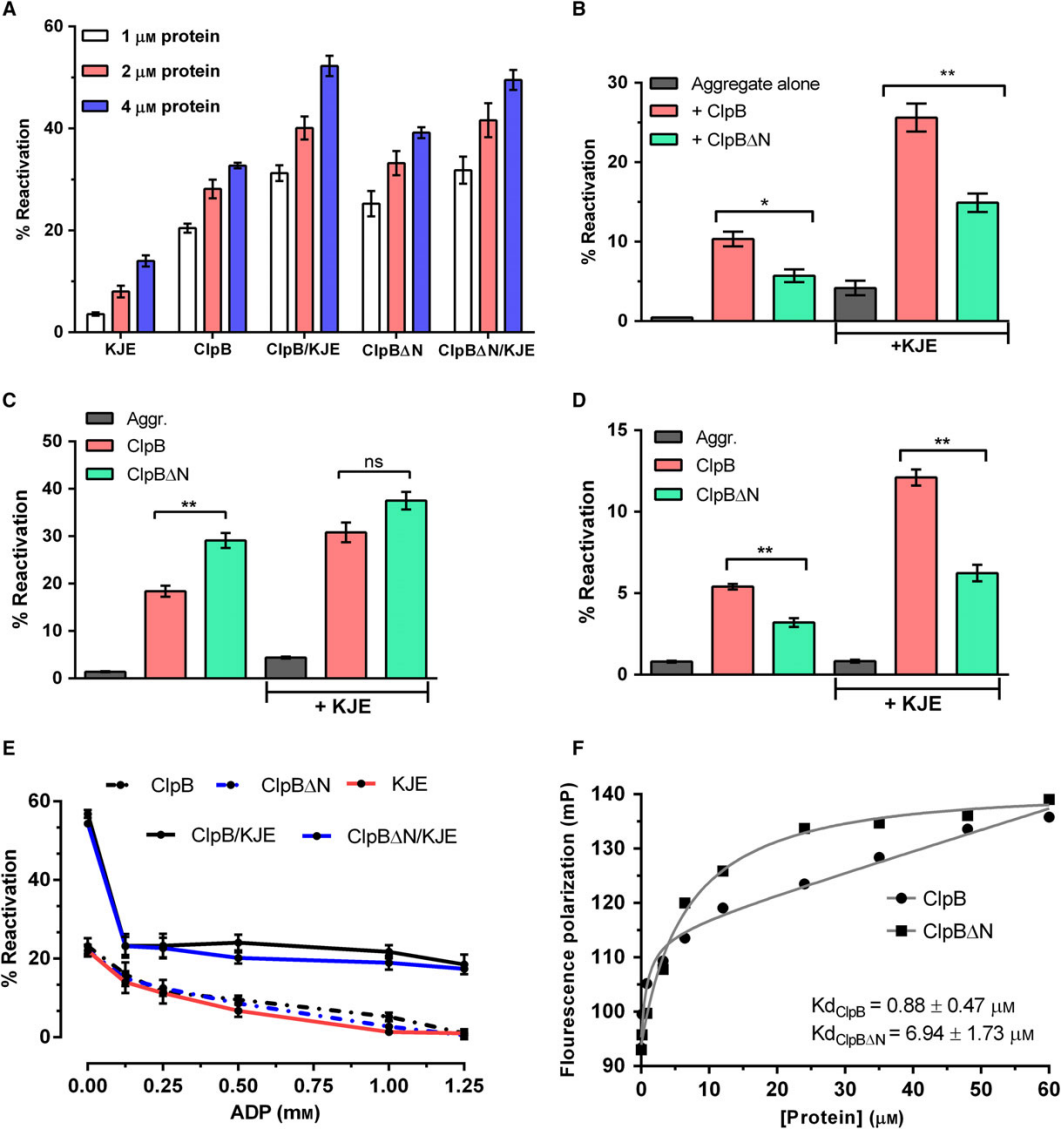
Resolubilization of aggregate and substrate binding ability of ClpB∆N *versus* ClpB. (A) Reactivation of small aggregates of MDH by ClpB or ClpB∆N, assayed in a dose‐dependent manner in the presence or absence of KJE chaperone complex. Protein concentration refers to the concentration of ClpB or ClpB∆N added in the reaction. (B) Reactivation of large aggregates of MDH by ClpB or ClpB∆N in the presence or absence of KJE chaperone complex. Spontaneous reactivation of MDH has been normalized from all the reactions. Activity recorded at the end of 1 h of assay is reported as the percentage activity of native MDH. *P < 0.05, **P < 0.001. (C,D) Reactivation of small or large aggregates of luciferase by ClpB (C) or ClpB∆N (D), respectively, in the presence or absence of KJE chaperone complex. Reactivation at the end of the assay is reported as percent luminescence of native luciferase. **P ≤ 0.005. (E) Effect of addition of ADP (0–1.25 mm) to the reactivation reaction mixtures containing MDH aggregates. Protein concentrations in the above reaction mixtures were 1 μm MDH or 0.1 μm luciferase, 2 μm ClpB or ClpB∆N, 1 μm DnaK, 0.5 μm DnaJ1 and 0.2 μm GrpE. ATP (5 mm) was added to all the reactions unless mentioned otherwise. The data represent mean ± SEM of three independent experiments done in triplicate using different preparations of substrate and protein each time. Statistical analysis was performed using Student's *t* test. (F) Substrate binding isotherms of ClpB *versus* ClpB∆N. Fluorescence polarization of 70 nm FITC–casein was recorded in the presence of varying concentrations of ClpB or ClpB∆N. Curve fitting and calculation of the dissociation constants were done using one‐site total binding equation in prism software. See ‘Materials and methods’ for the details.

To gain insight into the role of the NTD of *Mtb* ClpB in its association with the KJE chaperone complex and in resolving aggregates, we investigated the disaggregation activity of the two *Mtb* ClpB isoforms, using aggregated MDH as the substrate. For using as substrate, we generated MDH aggregates of two different sizes by heat‐aggregating them for different durations, as has been done earlier for glucose‐6‐phosphate dehydrogenase 40 and described in the ‘Materials and methods’ section. The size of the aggregates was validated by dynamic light scattering, which showed a size difference of ~ 100‐fold between the large and the small aggregates (Fig. S2). Firstly, we reconstituted the ClpB–KJE bi‐chaperone machinery and confirmed its functionality by following the reactivation kinetics of thermally aggregated MDH. As seen in Fig. S1C, ClpB and KJE could individually reactivate small aggregates of MDH to some extent, while in combination they displayed a remarkable increase in the reactivation activity. Subsequently, to test whether ClpB∆N behaved any differently from ClpB, small aggregates of MDH were incubated with increasing concentrations of ClpB or ClpB∆N alone or in combination with KJE for 1 h and solubilization of MDH was analyzed. At a concentration of 1 μm, ClpB alone was able to resolubilize about 20% of aggregated MDH, whereas ClpB∆N alone could resolubilize about 25% of the same (Fig. 7A). Addition of 1 μm KJE complex to the same reaction enhanced the reactivation activity of ClpB and ClpB∆N to 31 and 32%, respectively. The KJE complex alone showed a weak reactivation activity of 3.6%. The reactivation activity in all cases was found to increase in a dose‐dependent manner (Fig. 7A). It was evident from this result that ClpB∆N has comparable or moderately better disaggregation activity, and similar to ClpB, the addition of the KJE complex to ClpB∆N displayed enhancement suggesting that the NTD possibly has no significant influence on the interaction of ClpB with the KJE chaperone complex.

Further, to investigate whether the extent of aggregation or structural disorder of the substrates could also reflect on the reactivation activity of ClpB isoforms, the large aggregates of MDH were used as substrate. As done with the small aggregates, the disaggregation of the large aggregates was measured in the presence of ClpB or ClpB∆N alone, or in combination with the KJE complex. ClpB could resolve larger aggregates more efficiently compared to ClpB∆N and was found to have nearly 2‐fold higher reactivation activity (Fig. 7B). However, the reactivation activity dropped in general with all combinations compared to that observed for small aggregates in Fig. 7A. A drop of 50% was recorded in the reactivation activity of ClpB alone and 40% with KJE. In contrast, it decreased by 80% for ClpB∆N alone and 48% with KJE. These results indicate that the reactivation activity of ClpB variants is directly related to the size of aggregates.

To validate our findings from the MDH reactivation assay with another substrate, small and large aggregates of luciferase were made by thermal denaturation at 43 °C and their size was estimated using dynamic light scattering (Fig. S2). The reactivation of aggregates was monitored by recording luminescence. As seen with MDH, ClpB∆N performed better than ClpB in reactivating smaller aggregates of luciferase also, both alone as well as with KJE (Fig. 7C). However, it was found to be less efficient in reactivating larger aggregates of luciferase also and showed a reduction of more than 20% as compared to reactivation by ClpB alone and with KJE (Fig. 7D). Also, the reactivation efficiency was observed to have reduced greatly, upon increasing the aggregate size of luciferase, for all the combinations of chaperones. Taken together, these results imply that despite having structural and some functional properties akin to the full length ClpB protein, ClpB∆N does not interact that well with the highly aggregated substrates. In light of these results, it appears that *in vivo* the two isoforms might be catering to aggregates of different orders or even different client proteins. A functional overlap has been described by Doyle *et al*. 41 between the NTD and NBD1 of *E. coli* ClpB, where either of the two domains is shown to be essential for substrate priming and initial disaggregation. Thus, it is also possible that the *Mtb* ClpB NTD is also involved in initial extraction of large aggregates.

We also investigated the effect of ADP on the protein resolubilizing activity of ClpB and ClpB∆N. We found that, even under saturating levels of ATP (5 mm here), increasing concentrations of ADP significantly inhibited the reactivation of thermally aggregated MDH by ClpB or ClpB∆N (Fig. 7E). At a concentration of 1.25 mm, ADP completely inhibited the reactivation activity of both the ClpB isoforms. In the presence of DnaK there was a sharp decrease of about 40% in their reactivation activity at as low as 0.125 mm ADP, but it remained limited to ~ 40–45% for both the isoforms even at 1.25 mm ADP (Fig. 7E). Therefore, DnaK appears to help ClpB and ClpB∆N proteins in overcoming the inhibition posed by ADP.

### Loss of NTD results in decreased affinity for substrates

We next sought to quantitatively confirm whether the interaction between ClpB∆N and higher order aggregates was compromised. For this, we compared the substrate binding ability of ClpB∆N *versus* ClpB using fluorescence polarization. We used fluorescently tagged α‐casein as the substrate because the high specific optical rotation value of α‐casein indicates that it closely resembles proteins that are unfolded by guanidine 42. In addition, α‐casein is reported to bind *E. coli* ClpB with similar affinity as heat‐inactivated luciferase 17. ClpB homologs in other bacteria are known to have ATP‐dependent dynamic and transient interactions with their substrates 5, 34, 43, 44, 45. However, such interactions are difficult to detect by basic polarization experiment alone, and therefore in order to lock our proteins in substrate‐bound state we used 2 mm ATPγS instead of ATP. With FITC–casein, an increase in polarization signal against increasing concentrations of ClpB or ClpB∆N was observed (Fig. 7F), which reflects a successful interaction of both the proteins with the substrate. However, the dissociation constants for the two proteins differed appreciably, while ClpB was found to have a *K*_d_ of 0.88 ± 0.47 μm, the *K*_d_ for ClpB∆N was 6.94 ± 1.73 μm. This significant difference in the affinities for the same substrate argues that the NTD is indeed involved in stabilizing the interaction between *Mtb* ClpB and aggregates of higher order.

### The *Mtb* NTD contains a putative substrate‐binding groove of hydrophobic nature

**Figure 8:**
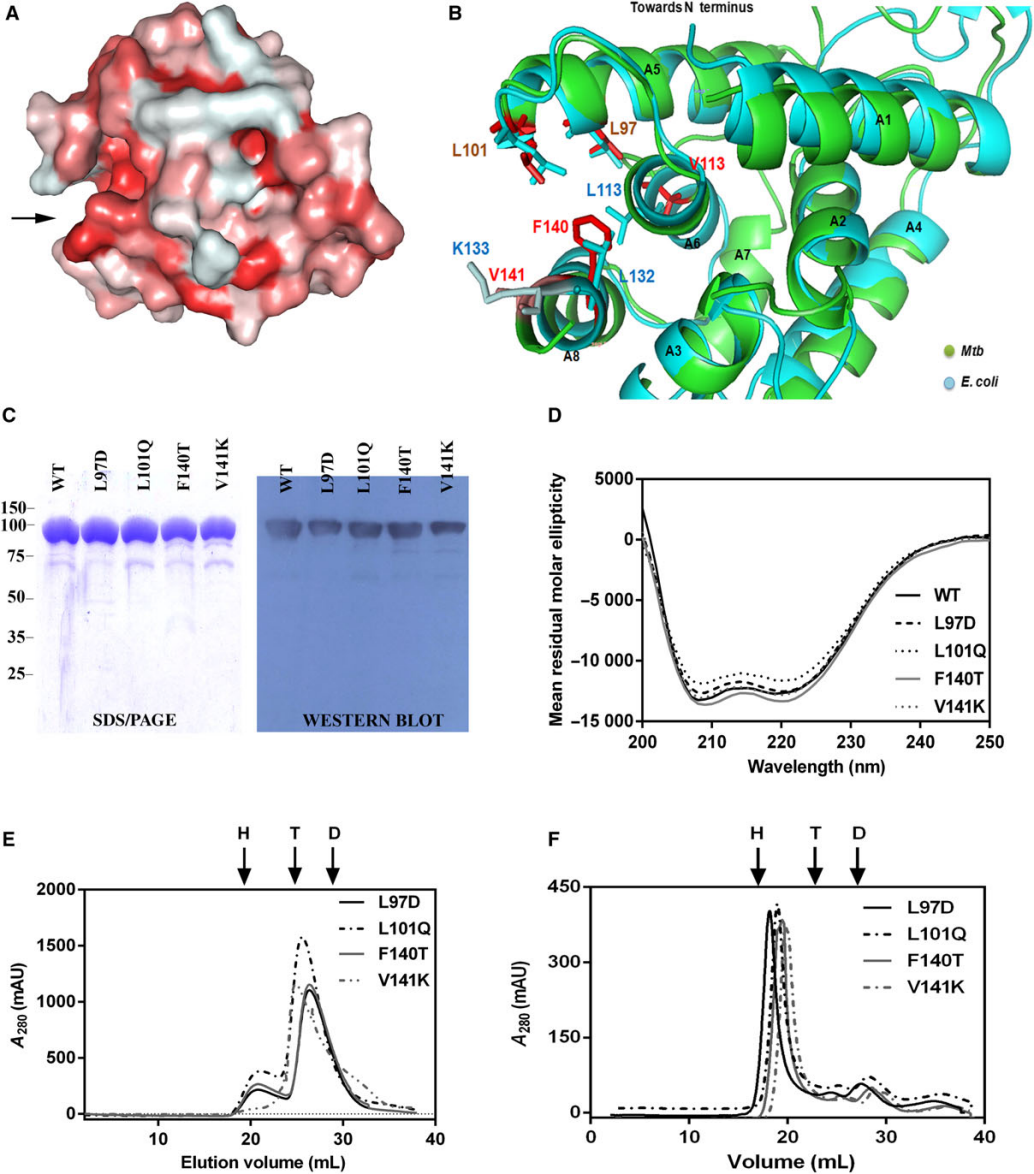
Characterization of the NTD of ClpB. (A) Surface view of the NTD of *Mtb* ClpB protein. The arrow at the left side marks the entrance to the hydrophobic groove. The homology model was built using the swiss‐MODEL workstation, with the structure of NTD of *E. coli* as template and pymol has been used to illustrate the surface view. The extent of hydrophobicity is shown by color gradient, red most hydrophobic and white least hydrophobic. (B) Cartoon view of the NTD of *Mtb* ClpB protein aligned against that of *E. coli* ClpB. Helices A1–A8 of *E. coli* NTD are labelled in black illustrating the presence of an internal pseudo 2‐fold symmetry in both the homologs. Some residues involved in forming the groove are highlighted as stick figures: *Mtb* 61 and *E. coli* (cyan). Conserved residues are shown in brown. Orientation of the groove is same as in (A). (C) 12.5% SDS/PAGE showing purified recombinant NTD mutants and the western blot alongside shows validation of the mutant proteins by probing against α‐ClpB antibody. (D) Secondary structure of the mutants compared with WT using CD spectroscopy in far‐UV region. (E,F) Oligomeric status of the mutants analyzed by gel filtration chromatography in the absence or presence of 5 mm ATP, respectively, using Superdex‐200 column. Arrows mark the position of elution of hexamer (H), tetramer (T) or dimer (D), which corresponds to the elution volume of molecular mass standards: thyroglobulin (660 kDa), ferritin (440 kDa), and aldolase (168 kDa). Figures are representative of experiments done at least twice.

Since the NTD of *Mtb* ClpB protein shows about 48% sequence similarity and 31% identity with that of the *E. coli* ClpB, we built a putative 3D model of the NTD with the swiss‐model tool, using the crystal structure of the NTD of *E. coli* (PDB no. 1KHY) as the template 46. It was further refined using the web server 3Drefine. The resulting homology model had a GQME and QMEAN *Z*‐score of 0.76 and −0.27 respectively, and r.m.s.d. of ~ 0.34 Å. The refined model was validated using the online tool procheck, and was found to be satisfactory. We found the surface of this domain to be hydrophobic in nature, consisting of an apparent groove largely made up of hydrophobic residues (Fig. 8A). Such a groove has also been reported to be present on the surface on the *E. coli* ClpB NTD 46. Structural alignment of the two homologous NTDs reveals that there is considerable similarity in the structures and symmetry of the two molecules. The crystal structure of the *E. coli* NTD shows it to comprise eight α‐helices, annotated as A1–A8 47, which were found to align with the eight α‐helices found in the *Mtb* NTD (Fig. 8B). The NTD of *Mtb* ClpB also possesses a similar internal pseudo 2‐fold symmetry. The helices A5, A6 and A8, which are a part of the C terminus of this domain, were found to constitute the hydrophobic groove. With the help of the pymol tool we identified the residues L97, L101, V113, F140 and V141 from each of the three helices A5, A6 and A8 to be located strategically in the groove such that they might be participating in the priming of substrate prior to its translocation to the central channel (Fig. 8B). Further, we chose the residues in helices A5 and A8 for our study since these were placed at critical entry points of the groove and would thus be the most probable candidates involved in initial interaction with the denatured polypeptides. The residues L97 and L101 from helix A5 were found to be conserved across species, while F140 and V141, from helix A8, were unique to *Mtb*. These four residues were mutated to amino acids with charged or polar side chains in order to disturb the hydrophobic nature of the groove, and four mutants, L97→D, L101→Q, F140→T and V141→K, were generated. The substitutions in all four point mutants were shown to have negligible effects on the secondary structure of the protein *in silico*. The mutants were generated by site‐directed mutagenesis and proteins were made as was done for the WT ClpB. The mutants reacted with anti‐ClpB antibody with the same affinity on a western blot as the WT (Fig. 8C). The CD spectra of the mutants in the far‐UV region were compared with that of the WT. All the mutants showed a CD spectrum similar to that of the WT, which shows proper folding of mutant proteins (Fig. 8D). Further, the elution pattern of mutants through the gel filtration column (Fig. 8E) was comparable to that observed for the WT ClpB eluted under the same conditions (Fig. 4A) suggesting that there were no conformational defects in the mutants. We also tested the propensity of mutant ClpB proteins to hexamerize in the presence of ATP (Fig. 8F) and found them to be similar to the WT ClpB (Fig. 4C).

### The putative hydrophobic groove is critical for stabilizing the interaction of ClpB with highly aggregated substrates

**Figure 9:**
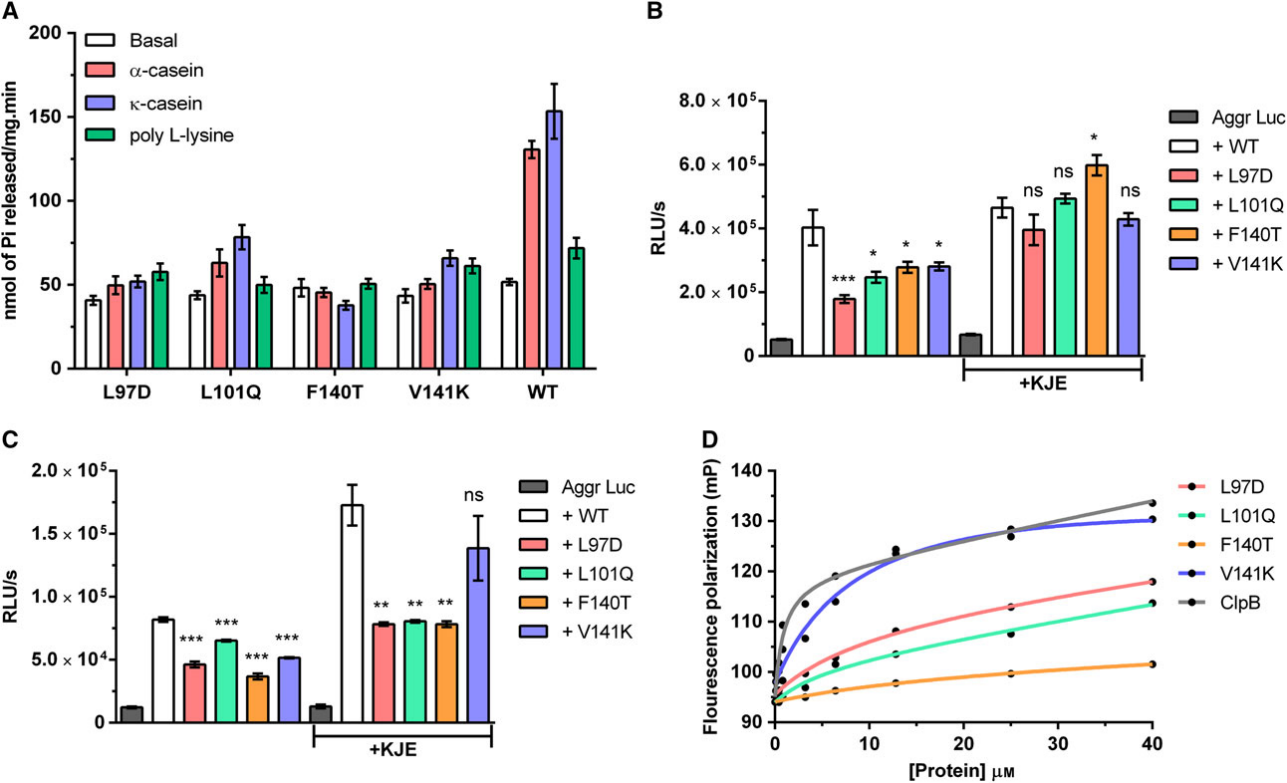
Comparison of the biochemical properties of the NTD mutants with WT ClpB. (A) Basal ATPase activity of mutants and its modulation by different substrates with respect to the WT ClpB. Substrate concentrations were same as shown in Fig. 4B. (B,C) Reactivation of small (B) and large (C) aggregates of luciferase by mutants in the presence or absence of KJE chaperones, respectively. Values are presented as relative luminescence units produced per second by each reaction (RLU/s). *P < 0.05, **P <* *0.005, ***P <* *0.0001. Protein concentrations were same as in Fig. 7C,D. The data represent mean ± SEM of three independent experiments done in triplicate. Statistical analysis was performed using one‐way anova. (D) Substrate binding isotherm of mutants compared to WT ClpB. Experiment and curve fitting were done as described earlier.

To investigate the effect of substitutions made in the hydrophobic groove on the chaperonic activities of ClpB, we functionally characterized the ClpB mutants. All four mutants were comparable to the WT in terms of their basal ATP hydrolyzing activity; however, unlike the WT, they exhibited severe defects in stimulation with intrinsically disordered substrates (Fig. 9A). The F140T mutant, in particular, showed no stimulation at all in its ATPase activity. The finding corroborates the previous result shown in this study (Fig. 5B), where loss of the NTD resulted in compromised stimulation of ATPase activity by substrates. Subsequently, we tested the protein disaggregation ability of NTD mutants using the luciferase reactivation assay. All the mutant ClpB proteins exhibited weak reactivation activity on small aggregates of luciferase by themselves, which ranged from 44 to 69% of the activity of WT ClpB, with L97D being the least active (Fig. 9B; Table 2). However, addition of KJE compensated for the loss in chaperonic activity (Fig. 9B; Table 2). Furthermore, F140T in conjunction with KJE showed an increase of ~ 28% in luciferase reactivation compared with WT KJE (Fig. 9B and Table 2). This indicates that although the hydrophobic groove mutants could not interact stably with aggregated luciferase on their own, complexing with KJE could offset the defect. As a follow‐up, we checked the efficiency of NTD mutants to disaggregate large aggregates of luciferase (Fig. 9C). We observed the basal reactivation activity of mutants to have decreased significantly to ~ 45–80% of that of the WT ClpB, with the L97D mutant being the least active of all. However, unlike in the case of small aggregates, the addition of the KJE complex could not compensate for the deficiency in reactivation of large aggregates of luciferase by L97D, L101Q and F140T mutants with the exception of V141K‐KJE, which acted better than other mutants and gained as much as 80% of the WT KJE activity (Fig. 9C; Table 2).

**Table 2:** Comparison of reactivation of aggregated luciferase by NTD mutants with WT ClpB. Recombinant firefly luciferase (Luc) was heat‐aggregated for 5 or 15 min to generate small or large aggregates, respectively. The aggregates were treated with WT or mutant ClpB and aliquots were taken for luciferase assay. The values represent luminescence recorded as RLU s^−1^ (relative luminescence units produced per second) and those in parenthesis represent the percentage of reactivation of aggregates. Data represent the mean ± SD of three independent experiments

Proteins	Luminescence (RLU s^−1^)			
	Small aggregates		Large aggregates	
	−KJE	+KJE	−KJE	+KJE
Luc alone	5.2 ± 0.3 × 10^4^ (13%)	6.7 ± 0.4 × 10^4^ (14%)	1.2 ± 0.1 × 10^4^ (15%)	1.3 ± 0.2 × 10^4^ (8%)
Luc + WT ClpB	4.0 ± 0.3 × 10^5^ (100%)	4.6 ± 0.5 × 10^5^ (100%)	8.2 ± 0.3 × 10^4^ (100%)	1.7 ± 0.3 × 10^5^ (100%)
Luc + L97D	1.8 ± 0.2 × 10^5^ (44%)	3.9 ± 0.8 × 10^5^ (85%)	3.6 ± 0.4 × 10^4^ (45%)	0.7 ± 0.02 × 10^5^ (45%)
Luc + L101Q	2.5 ± 0.3 × 10^5^ (61%)	4.9 ± 0.3 × 10^5^ (106%)	3.0 ± 0.1 × 10^4^ (37%)	0.8 ± 0.02 × 10^5^ (47%)
Luc + F140T	2.8 ± 0.9 × 10^5^ (69%)	6.0 ± 0.5 × 10^5^ (128%)	3.7 ± 0.4 × 10^4^ (45%)	0.9 ± 0.03 × 10^5^ (53%)
Luc + V141K	2.8 ± 0.2 × 10^5^ (70%)	4.3 ± 0.3 × 10^5^ (92%)	5.1 ± 0.08 × 10^4^ (63%)	1.4 ± 0.4 × 10^5^ (80%)

To establish whether the compromised chaperonic activity of hydrophobic groove mutants was indeed an implication of unstable substrate binding, we compared their binding isotherms with WT by fluorescence polarization using FITC–casein as the substrate (Fig. 9D). As seen from the binding isotherms of the mutants and their dissociation constant (*K*_d_) values (Fig. 9D; Table 3), none of the mutants could bind FITC–casein as well to as the WT. While all the mutants displayed deficiency in substrate binding, F140T was found to have the weakest affinity for FITC–casein. This result further confirms that the hydrophobic groove has a crucial role in engaging higher order protein aggregates in a stable manner for their disaggregation to follow.

**Table 3:** Comparison of substrate binding ability of NTD mutants with WT ClpB. Fluorescence polarization of 70 nm FITC–casein was recorded in the presence of mutants or WT ClpB protein. Apparent *K*_d_ values were calculated from the binding isotherms of respective proteins using the one‐site total binding model of prism software. Data represent the mean ± SEM of five independent values

Protein	*K*_d_ (μm)
WT	0.9 ± 0.3
L97D	10 ± 2.4
L101Q	5.3 ± 2.2
F140T	24 ± 7.3
V141K	10 ± 4.0

## Discussion

ClpB chaperone has been shown, in various organisms, to be involved in the disaggregation of protein aggregates formed as a result of stress 48. Intracellularly, ClpB is present in two forms, a WT and an N‐terminally truncated form lacking the NTD due to an internal translation start site. The significance and mechanism of function of the truncated isoform are not well understood. The current study envisaged investigating the mechanism of function of ClpB isoforms in *Mtb*, an important human pathogen. We structurally and functionally characterized the N‐terminally truncated variant of *Mtb* ClpB with respect to the full length ClpB in order to understand the significance of its NTD. We have also identified and delineated the involvement of some critical residues of the *Mtb* ClpB NTD in its substrate interaction.

The study shows that the NTD is involved neither in maintaining correct folding of the *Mtb* ClpB protein nor in the formation of stable hexamers. We have observed that, in general, both the isoforms of *Mtb* ClpB have a tendency to self‐associate, which is further enhanced under crowded conditions. This finding correlates with previous studies with homologs of ClpB of other organisms 49, 50. Stable hexamerization of *Mtb* ClpB is shown to be favored by ATP; however, ADP seems incompetent in this. This could be a consequence of the difference in the conformational states of the protein induced by ATP and ADP. We found the ATPase activity of ClpB∆N, the *Mtb* ClpB variant lacking the NTD, to be higher than that of the full length protein, which could be an indirect effect of changes in the pore shape leading to enhanced nucleotide binding. Further, this study shows that DnaK stimulates the ATPase activity of ClpB, but the effect is only moderate as DnaK homologs are reported to realize their full stimulatory potential in its aggregate bound state 51, 52. In addition, we also show that unstructured polypeptide substrates interact with the mycobacterial ClpB and differentially stimulate its ATPase activity. Contrary to this, ClpB∆N responds weakly to the stimuli of DnaK or the substrates. Previous studies have shown that DnaK and substrates of ClpB homologs potentiate its ATPase activity by causing large head to tail structural switches in the M‐domain leading to de‐repression of the oligomer and changes in its pore shape 53, 54, 55. As mentioned earlier, the pore shape of ClpB may change after losing the NTD, and it is likely that the molecule may achieve a state that cannot be further potentiated by DnaK or substrates. This re‐emphasizes the involvement of the NTD in perturbing conformational dynamics of the central pore.

We demonstrate that the affinity of *Mtb* ClpB for ADP is much higher than for ATP, and ADP considerably impedes both ATP hydrolysis and the reactivation activity of ClpB, even under saturating concentrations of ATP. Thus, the binding of ADP may lead ClpB to a rather repressed state. Our data show that the inhibitory effect of ADP could be alleviated to some extent by associating with DnaK, indicating that under such unfavorable physiological conditions ClpB isoforms manifest their optimal activity with the support of co‐chaperones. In the cellular environment, the inhibitory effect of ADP may play a significant part in the regulation and effective functioning of ClpB by possibly minimizing the consumption of energy by this chaperone machinery when not required and keeping a check on the otherwise potentially harmful remodeling activity of ClpB.

Further, we demonstrate that both the mycobacterial ClpB isoforms exert chaperonic activity to protect cellular proteins. We also report a prevention of aggregation activity of *Mtb* ClpB isoforms, by virtue of which they prevent thermal aggregation of luciferase, suggesting another role for these proteins. It is likely that the prevention of aggregation activity takes care of the *de novo* protein translation in the *Mtb* bacilli persisting under stressful conditions, by coating nascent polypeptide chains and preventing them from entering into non‐native conformations. Our results show that both ClpB and ClpB∆N exert their optimal prevention activity only in the ATP‐bound state, as even the active hexamers of ClpB isoforms, formed due to self‐association at higher concentrations, were deficient in preventing thermal aggregation of luciferase. However, the hydrolysis of ATP was not necessary for this. It therefore appears that the binding of ATP induces changes in the conformation of ClpB and ClpB∆N conducive to accessibility to substrates.

The study also demonstrates that the reactivation activity of both ClpB and ClpB∆N depends on the extent of aggregation of client proteins. We found ClpB∆N to be significantly less efficient in resolving higher order aggregates, which is a result of a severe defect in the substrate binding ability of the protein, after losing the NTD. It is likely that in the absence of any known signal peptide recognition mechanism, ClpB recognizes its clients by making contacts with the exposed hydrophobic patches on the surface of non‐natively folded proteins. It appears that the loss of the NTD adversely affects this process. To further address this, we studied the structural features of the *Mtb* ClpB NTD and compared them with the already known structure of the *E. coli* ClpB NTD. The two NTDs share most of the structural features and a hydrophobic groove on their surface 56, 57. The presence of a hydrophobic groove has also been acknowledged in *T. thermophilus* ClpB by Rosenzweig *et al*. 56. Based on our model, it appeared that the helices A5, A6 and A8 in the hydrophobic groove of *Mtb* ClpB might be the site of substrate interaction, and therefore we mutated four putatively critical residues, L97, L101, F140 and V141, located at the entry points of this groove to disturb its hydrophobicity. These mutations disrupted binding with the substrate leading to ineffective remodeling of the strongly aggregated ones. The conformation, oligomeric status and ATPase activity of the mutants were found to be intact and also comparable to those of the WT. However, the substrate‐dependent enhancement in ATPase activity was diminished and was found to be even less than that observed for ClpB∆N. The binding isotherm of the mutants confirmed that the hydrophobic groove is responsible for stable substrate interaction and initial substrate engagement prior to its translocation to the central channel. The NTD might in fact be blocking the entrance to the central channel and, as observed for ClpB∆N, deletion of the NTD releases the blockage such that the basal ATPase activity of ClpB∆N is potentiated. In addition, our observations from the expression profile of ClpB and ClpB∆N in *Mtb* under normal conditions and stress are indicative of a concerted mechanism of action of ClpB isoforms to deal with stress and point towards an important regulatory role of ClpB∆N protein.

Integrating our findings with previous studies on ClpB homologs, we propose a concise model of action of *Mtb* ClpB elucidating the role of its NTD. The core domains of ClpB, i.e. NBD1, MD and NDB2, adapt a double ringed hexameric conformation while the NTD of each protomer is loosely associated through a flexible linker. The NTD in this way acts as a checkpoint and controls access to the central channel of the core ring, which keeps the double tier in a semi‐repressed state or the state of its basal activity until its interaction with DnaK. The hydrophobic groove in the NTD of mycobacterial ClpB contacts the exposed hydrophobic patches on the substrate and primes it for threading through the central channel. The hydrophobic groove identified in this study possibly acts in a ‘capture and release’ manner causing minimal expulsion of rather large aggregates from the central channel. The core double ring of *Mtb* ClpB, similar to its homologs, acts in a rather wave‐like fashion that contracts and expands depending on its nucleotide state to translocate the substrate further. Taken together, our findings imply that the NTD plays a regulatory role in the substrate remodeling activity of ClpB by firstly blocking the entrance to the pore loops so that harmful over‐potentiation of the machinery can be restricted and secondly acting as an additional anchor for rather large aggregates. Our proposed model of *Mtb* ClpB action correlates with recent studies on *T. thermophilus* and *E. coli* ClpB that support the regulatory role of the NTD in transferring substrate to the ClpB pore 56, 58.

In conclusion, the present study demonstrates that the two isoforms of ClpB in *Mtb* can exert chaperonic activity and remodel aggregated protein substrates in conjunction with the KJE chaperone complex, but with differential efficiencies. The NTD of *Mtb* ClpB harbors a hydrophobic groove that is crucial for stable substrate binding and has to be maintained in order to perform the disaggregation of larger aggregates.

## Author contributions

PT and JKB conceived the study. PT, PP and VKP performed the experiments; PT and JKB analyzed the data and wrote the manuscript.

## Supporting information


**Fig. S1.** Characterization of DnaKJE proteins. (A) 12.5% SDS/PAGE showing purified recombinant DnaK, DnaJ1 and GrpE proteins of *Mtb* used in this study; numbers on the left of the image indicate molecular masses in kDa. (B) Stimulation of DnaK ATPase activity by varying amounts of DnaJ1. The specific ATPase activity is reported as nmol of P_i_ released per milligram of protein. (C) Measurement of the refolding activity of ClpB with or without KJE. The data represent mean ± SEM of three independent experiments done in triplicate.**Fig. S2.** Average size of the aggregates. The average hydrodynamic radius of the small and large aggregates of MDH and luciferase estimated by dynamic light scattering. The experiment was done in triplicate and each replicate was scanned 10 times. Values represent the mean of the triplicates and error bars represent the SEM.
