# Supplementary material for: The amino‐terminal domain of Mycobacterium tuberculosis ClpB protein plays a crucial role in its substrate disaggregation activity
Source: FEBS Open Bio. 2018 Sep 15;8(10):1669–90. doi: 10.1002/2211-5463.12509 (PMC6168691; doi:10.1002/2211-5463.12509)
Supplement: Supplementary file 1 — Fig. S1. Characterization of DnaKJE proteins. (A) 12.5% SDS/PAGE showing purified recombinant DnaK, DnaJ1 and GrpE proteins of Mtb used in this study; numbers on the left of the image indicate molecular masses in kDa. (B) Stimulation of DnaK ATPase activity by varying amounts of DnaJ1. The specific ATPase activity is reported as nmol of Pi released per milligram of protein. (C) Measurement of the refolding activity of ClpB with or without KJE. The data represent mean ± SEM of three independent experiments done in triplicate.Fig. S2. Average size of the aggregates. The average hydrodynamic radius of the small and large aggregates of MDH and luciferase estimated by dynamic light scattering. The experiment was done in triplicate and each replicate was scanned 10 times. Values represent the mean of the triplicates and error bars represent the SEM. [file FEB4-8-1669-s001.pdf]

FIGURE S1

A.

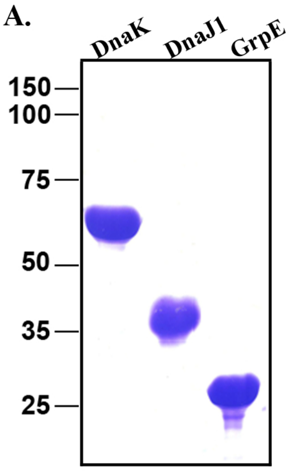

B.

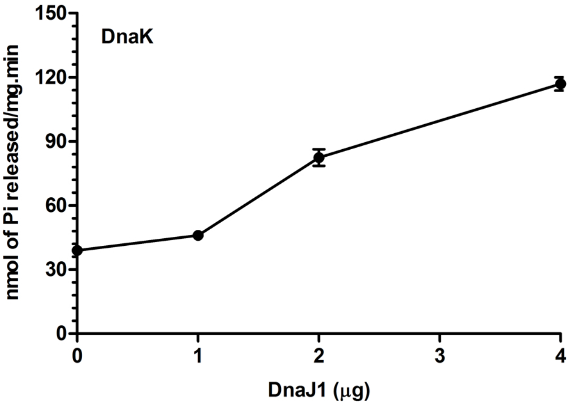

C.

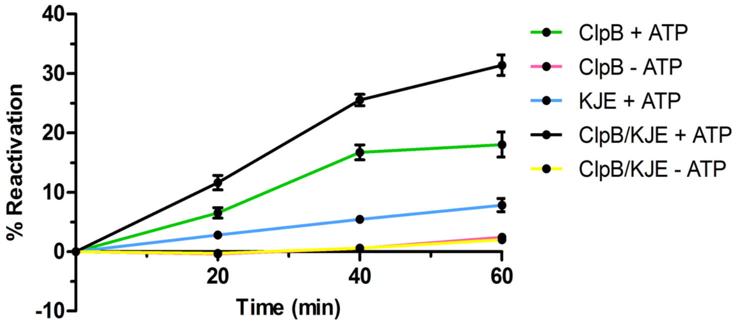

Figure S1. Characterization of DnaKJE proteins. *A*, 12.5% SDS-PAGE showing purified recombinant DnaK, DnaJ1 and GrpE proteins of *M. tb* used in this study; numbers on the left of the picture indicate molecular mass in kDa. *B*, Stimulation of DnaK ATPase activity by varying amounts of DnaJ1. The specific ATPase activity is reported as nmol of Pi released per minute per mg of protein. *C*, Measurement of the refolding activity of ClpB with or without KJE. The data represent mean ± SEM of three independent experiments done in triplicates.

FIGURE S2

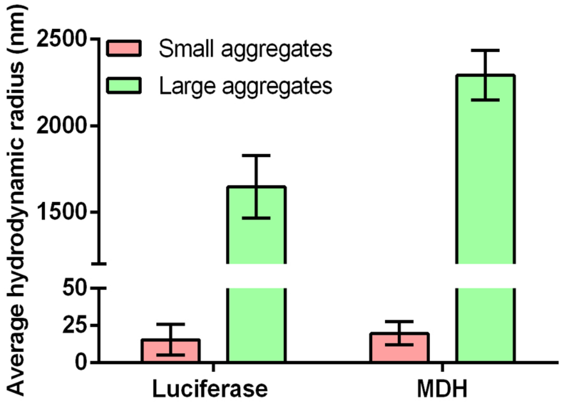

Average hydrodynamic radius (nm)
2500
2000
1500
50
25
0
Small aggregates
Large aggregates
Luciferase
MDH

Fig. S2. Average size of the aggregates. The average hydrodynamic radius of the small and large aggregates of MDH and luciferase estimated by dynamic light scattering. The experiment was done in triplicate and each replicate was scanned 10 times. Values represent the mean of the triplicates and error bars represent the SEM.
